# Ensuring Sustainable Development in Ghana: Public Health Security and Policy Concerns

**DOI:** 10.18502/ijph.v49i8.3909

**Published:** 2020-08

**Authors:** Abraham ASSAN, Ali AKBARI SARI, Amirhossein TAKIAN

**Affiliations:** 1. Department of Health Economics and Management, School of Public Health, Tehran University of Medical Sciences, Tehran, Iran; 2. Global Policy and Advocacy Network (GLOOPLAN), Accra, Ghana; 3. Department of Global Health and Public Policy, School of Public Health, Tehran University of Medical Sciences, Tehran, Iran; 4. Health Equity Research Centre (HERC), Tehran University of Medical Sciences, Tehran, Iran

## Dear Editor-in-Chief

Despite its perceived benefits, globalization has deepened health threats across borders (1). Such a situation has transformed heath into a multifaceted and transnational phenomenon, whose challenge is beyond the capacity of independent institutions and countries to address. Major political or economic unions like the G7 and G20 have strengthen both their interests and commitment to take up the issue of global health agenda more progressively and actively (2).

The Economic Community of West African States (ECOWAS) remain a significant socio-economic and political space in West Africa. Joined as a single body in 1975 by fifteen-member countries of West Africa, the club has made meaningful contributions to its member states’ sustainable development, particularly in the areas of energy supply, job creation, political and military assistance, and conflict resolution (3). Nevertheless, the role of ECOWAS in global as well as regional governance for public health security is understated.

Health security is primarily the protection from threats to health (4, 5). Infectious diseases continue to renew the importance of reducing the vulnerability of societies to health threats which spread across national boundaries., and the need of individual states to act effectively to contribute to public health security (6).

The ECOWAS platform has the capacity, we envisage, to serve as a policy window for member states to initiate sustainable mechanisms to improve regional public health security. While the conventional role of global powers (such as the United States) towards health in is fading, it is a right time to call on member states of ECOWAS to enhance their commitment to ensuring public health security in the region. Ghana, compared with many African States, maintains a key position in a “rising Africa”. This is as a result of its political stability (peaceful transition of political power), promising economic indicators, and its friendly relations with other nations. (7). As such, we advocate for Ghana to perform the frontline role in public health security of the region – although that might require considerable amount of resources from the government.

Within the current multifaceted and dynamic global context, tied with the never-ending cross-border movements of goods and people in the region, regional health initiative is required to prevent the Ghanaian society from various outbreaks, e.g. Ebola. Notwithstanding the above, policy incoherencies and lack of effective inter and intra institutional coordination at both national and regional levels, may undermine the multisectoral collaboration required between institutions like the Ministry of Health, the National Health Insurance Authority, the Ministry of Education, Immigration and the Police Department, Ministry of Labour, Ministry of Foreign Affairs and the Ministry of Trade and Industries, to boost response capacity in handling disease outbreak and emergencies in Ghana, and beyond.

The West African region will remain vulnerable to health risks – fascinated by its long-lasting integration development. In addition to individual member states’ concerns about their citizens’ health security, it will be of prudent self-interest for all states to also improve multilateral cooperation to shape regional governance for health, through robust joint diplomatic efforts.

We envision, Ghana, a member state of the ECOWAS, can take such a leading role to move regional health into the scope of high politics. To accomplish this mission, Ghana needs to first reinvigorate its national health system in line with the requirements of the 2030 Sustainable Development Goals in Africa, and to include: capacity building of national policy makers on global health diplomacy and global health governance; build a research culture that draws on the practicality of health diplomacy; improve the competencies of diplomats and their role in fostering government’s international influence in matters of public health security and its relevance to the region; and liaise with other nations and institutions to help mobilize resources to pursue a collective fight for global health agenda.
